# Supplementary material for: Promising Phytogenic Feed Additives Used as Anti-Mycotoxin Solutions in Animal Nutrition
Source: Toxins (Basel). 2024 Oct 10;16(10):434. doi: 10.3390/toxins16100434 (PMC11511298; doi:10.3390/toxins16100434)
Supplement: Supplementary file 1 [file toxins-16-00434-s001.zip › toxins-3211547-supplementary.pdf]

Supplementary Materials: Promising phytogenic feed additives used as anti-mycotoxin solutions in animal nutrition

Supplementary Table S1. Studies with phytogenic interventions targeting different livestock, aquaculture, and pet species challenged by mycotoxins. CUR, curcumin; TP, turmeric; HMB, Herbal Mycotoxin Binder containing curcuminoids; Syl, silymarin; AF, aflatoxin; Fs, fumonisins; ZEN, zearalenone; OTA, Ochratoxin A; DON; deoxynivalenol; GSE, grape seed extract; GSPE, grape seed proanthocyanidin extract; PDOP, pectin derived from orange peel; LYC, lycopene; CAT, catalase; SOD, superoxide dismutase; GPx, Glutathione peroxidase; GSH, Glutathione; GST, Glutathione S-Transferase; ROS, reactive oxygen species; MDA, Malondialdehyde; TBARS, Thiobarbituric acid reactive substances; WG, weight gain; FCR, Feed conversion ratio; ADG, Average daily gain; FI, Feed intake; Chol, cholesterol; trigl, triglycerides; RLW, relative liver weigth; 8-OHdG, 8-Hydroxy-2'-deoxyguanosine; LPS, Lipopolysaccharide.

Target Species	Study Characteristics	Mycotoxin (dose)	Phytogenic (dose)	Phytogenic's Effects	References
**Curcumin**					
Broiler chickens	Male, Ross 308, 18-days-old (*n* = 32)	0,02 mg/kg feed AFB1	400 mg/kg feed CUR	Attenuation of oxidative stress parameters (SOD, CAT, and GPx activities, 8-OHdG & MDA levels) in kidneys.	[95]
Broiler chickens	Male, ROSS 308, 18-days-old (*n* = 32)	0,02 mg/kg feed AFB1	400 mg/kg feed TP	Reduced lipid peroxidation and oxidative, restored CYP2A6 and Nrf2 expression and reduced AFB1 residues levels in the liver.	[96]
Broiler chickens	Male, Arbor Acres, 180-day-old (*n* = 96)	1 mg/kg AFB1	444 mg/kg curcuminoids & 300 mg/kg CUR	Regulation of TLR4/RIPK signaling pathway, reduction of oxidative stress biomarkers and inflammatory cytokines levels, and attenuation of necroptosis and inflammation gene expression in the liver.	[78]
Broiler chickens	Male, Ross x Ross, 8-day-old (*n* = 30)	1 mg/kg AFB1	74, 222 & 444 mg/kg TP	Amelioration of performance parameters (RLW, WG, FCR) and serum total protein levels, AST, albumin, globulin and g-glutamyl transferase activity. Increase in antioxidant activity in the liver.	[97]
Broiler chickens	Male, Arbor Acres, 1-day-old (*n* = 320)	1 mg/kg AFB1	500 mg/kg CUR	Protective effect against damage to the liver and intestine through the Nrf2 signaling pathway. Improved intestinal permeability and oxidative stress. Amelioration of growth performance.	[99]
Broiler chickens	Arbor Acres, 1-day-old (*n* = 120)	5 mg/kg AFB1	150, 300 & 450 mg/kg	Alleviation of oxidative stress, and decreased the mRNA expression of CYP450 enzymes in duodenum. Amelioration of duodenal Abcb1 expression, P-glycoprotein (P-gp) level, and ATPase activities.	[91]
Broiler chickens	Male, 1-day-old (*n* = 120)	100 µg/kg AFB1	150 mg/kg CUR	Alleviate AFB_1-induced liver injury. Suppression of serum biochemistry changes and histopathological lesions by increasing	[100]

				antioxidant capacities and inhibiting the regulatory role of CYP450.	
Broiler chickens	Ross strain, 1-day-old (*n* = 270)	3 ppm AFB1	0,05% CUR	Reduction of ALT, AST, and uric acid levels. Increased total protein, calcium, and HDL values and reduced uric acid.	[101]
Broiler chickens	Male, Cobb500, 1-day-old (*n* = 50)	600 mg/kg FB1	50 mg/kg CUR , 5 and 10 mg /kg feed of nanocurcumin	curcumin nanocapsules at 10 mg/kg had hepaprotective and antioxidant effects (lower levels of hepatic ROS and TBARS), as well as causing small improvements in the weight of the chicks.	[106]
Broiler chickens	one-day-old (*n* = 24)	1 mg/kg OTA	400 mg/kg CUR	In the liver and kidneys, downregulation of GPx4, KEAP1, NRF2, and AHR transcription factors. In kidneys, upregulation of NRF2 gene. CURC antioxidant response is regulated through the KEAP1-NRF2-ARE and AHR pathways.	[107]
Broiler chickens		0,5 ppm OTA	2 g/kg feed CUR	CUR reduced the severity of toxicity in kidneys	[108]
Ducks	Male, 1-day-old (*n* = 450)	60 μg/kg AFB1	500 mg/kg CUR	Ameliorates acute liver lesions by inhibiting the NLRP3–caspase-1 signaling pathway. Upregulates NrF2 and antioxidant-related gene expressions, inhibits CYP1A4, and increases the antioxidant capacity.	[102]
Ducks	1-day-old (*n* = 40)	0,1 mg/kg AFB1	400 mg/kg CUR	Ameliorates histological ileum injuries and intestinal flora disturbance by modulating both the NLRP3 inflammasome and the TLR4/NF-κB signaling pathway.	[103]
Ducks	Mixed-sex, White Pekin, 1-day-old (*n* = 720)	2 mg/kg feed OTA	400 mg/kg CUR	Alleviates the upregulation of inflammation and oxidative stress pathways, and decreases serum LPS content. Decreases the expression of *TLR4 and* regulates gut microbiota reducing inflammation in the intestine.	[81]
Ducks	Mixed-sex, White Pekin, 1-day-old (*n* = 540)	2 mg/kg feed OTA	400 mg/kg CUR	Lowers lipid peroxidation, improves intestinal barrier and mitochondrial function, mainly by affecting tight junctions, cytoskeleton proteins, apoptotic proteins, and mitochondrial transcription factors. Alleviates destruction of the intestinal barrier	[109]
Egg-laying hens	28-week-old (n = 36)	500 μg/kg AFB1	0,2% HMB	Restoration of feed consumption and egg production.	[104]
Layer Japanese quails	45-week-old (n = 220)	1,5 ppm AFB1	0,03% HMB	Enhance egg production, weight, eggshell, biochemical parameters (Chol, Trigl, ALT, ALP, protein), and modulate gut physiology and microbiota.	[105]
Swine	Porcine PK-15 cell line	OTA (0,5–10 μM), FB1 (10–100 μM), DON (0,01–2.5 μM)	1-10 μM CUR	Enhances the viability of cells exposed to the mycotoxins and attenuates ROS formation by FB1 and DON. Decreases apoptosis in cells exposed to DON.	[110]

Swine	Mixed-sex Pigs (*n* = 120)	2,5 mg/kg DON	0.2% Phytobiotic additive containing CUR	Enhances the growth performance and shows the potential to enhance the immune response in pigs.	[111]
Swine	Large White × Landrace (DanBred), Weaned Piglets, 28-day-old (*n* = 150)	Multi-contaminated feed (Farm 1: FB1, FB2, ZEN and T-2 toxin Farm 2: FB1, FB2 and AFB1)	2,5 kg/t multi-component mycotoxin-detoxifier (with CUR)	Antioxidant activity is increased. CUR improves BW and ADG, and mortality. Liver and intestinal damage were reduced significantly	[112]
Swine	Large White × Landrace, DanBred Sows, 366-day-old (*n* = 80)	Multi-contaminated feed (Farm 1: FB1 and FB2 Farm 2: FB1, FB2 and AFB1)	1,5 & 2,5 kg/t multi-component mycotoxin-detoxifier (with CUR)	Ameliorated reproductive parameters (mammary gland state) and litter characteristics. Antioxidant activity is increased.	[113]
Bovine	bovine fetal hepatocyte-derived cell line (BFH12)	3,6 μM AFB1	10 μM CUR	Antioxidant players MDA concentration and NQ01 enzyme activity were increased, and CYP3A activity, which plays a role in the bioactivation of AFB1, was reduced.	[114]
Bovine	Dairy Cows, Holstein–Friesian, late lactation period (*n* = 8)	5 μg/kg AFB1	20 g TP dissolved in linseed oil/head/day	The low availability of curcumin in this formulation did not impact milk yield, milk composition, or somatic cell count.	[115]
Grass carp	Juvenile Grass carp (*n* = 720)	1,2 mg/kg OTA	400 mg/kg CUR	Ameliorates muscle toxicity and development, and enhances growth performance mainly by increasing the weight gain percentage and final body weight. Upregulates S6K1 and TOR, associated with the AKT/TOR signaling pathway.	[117]
Grass carp	Juvenile Grass carp (*n* = 180)	1,2 mg/kg OTA	400 mg/kg CUR	Inhibition of OTA absorption while promoting efflux transporters mRNA expression. Enhancement of the physical barrier by fortifying antioxidant defenses, reducing apoptosis, and strengthening tight junctions. Improvement of the chemical barrier by elevating goblet cells, immune components, and antimicrobial peptides. Enhances the immune barrier by suppressing TLR4 and NF-κB signaling pathways.	[118]
Grass carp	Juvenile Grass carp (*n* = 720)	1,2 mg/kg OTA	400 mg/kg CUR	Inhibits absorption and promotes efflux transporters mRNA expression, reducing the residuals of OTA. Decreases oxidative stress and enhances the expression of antioxidant enzymes. promote mitochondrial fusion proteins to inhibit the expression of mitotic proteins and mitochondrial autophagy	[119]

				proteins and enhance mitochondrial function. Reduces necroptosis-related gene expression. Reduces the expression of pro-inflammatory factors by inhibiting the TLR4/NF-κB signaling pathway.	
Red Tilapia	Fingerlings of O. niloticus (*n* = 240)	0,2 mL of *A. flavus* (4 × 10^3 conidia/mL)	40, 50 and 60 mg/kg nanocurcumin	Amelioration of hepatic damage and inflammation, intestinal dysfunction and morphology, splenic tissues and normal melano-splenic inflammatory macrophages, and improvement of the primary epithelium of gill lamella and secondary filament in gill tissue. Improves growth performance (increases BW, ADG, FI, fish survival, and decreases FCR). Ameliorates body composition by reducing lipid content and increasing protein content.	[120]
Nile Tilapia	Fingerlings of O. niloticus (*n* = 180)	6 mg/kg b.w. AFB1	10 and 20 g/kg diet CUR	Reduces activity of plasma AST, ALT, creatinine and uric acid values, and hepatic MDA. Increases plasma total protein and hepatic TAC activity.	[121]
Nile Tilapia	Fingerlings of O. niloticus (*n* = 180)	6 mg/kg b.w. AFB1	10 and 20 g/kg diet CUR	Hepatic lesions were reduced with CUR.	[122]
Nile Tilapia	Fingerlings of O. niloticus (*n* = 160)	2 ppm AFB1	5 g/kg CUR	Improves final body weight, ADWG and feed conversion ratio. Increases total protein levels and reduces transaminases, creatinine and uric acid. Reduces aflatoxin residues.	[123]
Shrimp	Juvenile Pacific white shrimp (Litopenaeus vannamei) (*n* =	500 µg/kg AFB1	100 and 200 mg/kg Zn-CUR	Ameliorates the toxic effects of AFB1 on growth performance. Enhances phenoloxidase (PO) ($P < 0.05$) activity. Modulates the antioxidation, immunological capacity and hepatoprotective of shrimp against AFB1.	[124]
Rabbit	Male, New Zealand rabbits, 6-8-wee-old (*n* = 35)	50 ug dissolved AFB1 in 0.5 ml of olive oil/ animal	15 mg/kg b.w CUR	Immune strengthening effect and protection of lipids and proteins from oxidative damage. Possesses a hepato-protective effect through scavenging of free radicals, or by enhancing the activity of antioxidants, which then detoxify the free radicals. Inhibitory action on biotransformation of AFB1 to their active epoxide derivatives. reduction in the incidence of coagulative necrosis of renal tubular lining epithelia.	[125]
**Silymarin**					
Broiler chickens	Male, 14-day-old (*n* = 21)	0,8 mg/kg of feed AFB1	Syl phytosome at 600 mg/kg b.w.	Improves ALT concentration, liver histology, feed intake, and BW gain.	[139]
Broiler chickens	Male, Ross 308, 1-day-old (*n* = 216)	250 and 500 ppb AFB1	0,5, and 1 % Syl seeds	Decreases albumin, direct bilirubin, calcium, and phosphorus levels.	[140]

Broiler chickens	Ross 308, 7-day-old (*n* = 336)	0,5 and 2 ppm AFB1	500 and 1000 ppm Syl	Increases in ADFI and ADWG, and improved FCR values. Suppresses ileal populations of Escherichia coli, Salmonella, Klebsiella, and total-negative bacteria. Increases villi height and VH:CD.	[141]
Broiler chickens	Cobb 500, 1-day-old (*n* = 240)	0,05 ppm AFs & 20 ppm Fs	100 mg/kg of feed Syl	Increases serum globulin concentration and reduced albumin concentration and ALT and AST serum activities Improves meat quality. reduces impairment of growth performance at the end of the productive cycle, prevents oxidative stress, and increases polyunsaturated fatty acids	[142]
Broiler chickens	Ross 308, 1-day-old (*n* = 160)	8,4 ppb Afs + 24,3 ppb T-2 toxin	0.5% Syl/ kg feed	Improves growth performance by ameliorating body weight, weekly weight gain, feed consumption, and feed conversion ratio. Increases the number of RBCs, WBCs count, PCV ratio, Hb concentration.	[143]
Broiler chickens	Male, Ross 308, 1-day-old, (*n* = 144)	3,0 mg/kg OTA	1% Syl	Improves glucose, uric acid, AST and ALT levels. Reverts pathomorphological changes in kidneys and liver.	[147]
Broiler chickens	Male, Ross 308, 24-day-old (*n* = 220)	500 ppb AFB1	0,5% Syl seeds	Decreases serum AST and LDH levels and increases albumin. Improves GGT activity and total protein levels.	[156]
Duck	Female, white Hungarian ducks (*n* = 18)	4,9 mg/kg DON + 0,66 mg/kg ZEA	0,5% Syl seed/ kg feed	Enhances the antioxidant defense by the elevation of sulfhydryl groups concentration and reducing power property resulted in decreased total scavenger capacity. Reduces lipid peroxidation, and improves histological changes.	[144]
Duck	Female, white Hungarian ducks, 1-day-old (*n* = 80)	4,9 mg/kg DON + 0,66 mg/kg ZEA	0,5% Syl seed/ kg feed	Decreases the severity of solitary cell death and infiltration of lympho- and histiocytes in the liver. Prevents lymphocyte depletion in the spleen and bursa of Fabricius	[145]
Laying Hens	Inshas, local layer hens, 28-week-old (*n* = 300)	1ppm OTA	1000 mg/kg feed Syl	Increases albumin, globulin, serum total proteins, total erythrocytes count, leukocyte count, PCV, and Hb. Decreases ALT and AST, creatinine and uric acid. Confers hepatoprotective effects.	[146]
Japanese quail	Mixed-sex, Coturnix coturnix japónica, 7-day-old (*n* = 1200)	2,2 mg/kg AFs	1000 and 2000 mg/kg Syl	Increases DWG and EPI and reduces feed conversion ratio. Reduces AST, ALT, and ALK activity. Alleviates performance parameters.	[148]
Swine	Male, Weaned Piglets, Large white × Landra ce, 26-day-old (*n* = 72)	500 ppb AFB1 and AFB2	1000 mg/kg anti-mycotoxin blend with Syl (2,75 g)	Protects health and minimizes the negative effects of aflatoxin on weight gain. Reduces AST and ALT levels, monocytes in the blood, hepatic GST activity and ROS levels in the liver and spleen.	[149]

Swine	Mixed-sex, Weaned Piglets, 25-day-old (*n* = 112)	0.35 mg/kg feed ZEN and 0.5 mg/kg feed T-2 toxin	3 g/kg feed anti-mycotoxin blend with Syl	Induces dose-dependent reduction of genital organs (sum of ovaries, cornu uteri and vagina-vestibule) relative weight and vulva size. Reduces residue levels of ZEN in the liver and kidneys. Reduces T-2 residues in kidneys.	[150]
Swine	Weaned pigs, 28-day-old (*n* = 60)	992 µg ZEN/kg feed and 531 µg OTA/kg	1,5 and 3 g/kg feed anti-mycotoxin blend with Syl	Improves FCR, reduction of OTA, ZEN and a-ZEL residues in the liver.	[151]
Swine	Mixed-sex, weaned pigs (Topigs Norsvin), 24-day-age (*n* = 90)	PCH= DON: 4,500 µg/kg; ZEA: 500 µg/kg; FB: 18,000 µg/kg PCL= DON: 900 µg/kg; ZEA: 100 µg/kg; FB: 5,000 µg/kg	5 kg/ton anti-mycotoxin blend with Syl	Provides the ability to improve the performance parameters affected by mycotoxins. Improves antioxidant balance.	[152]
Bovine	Bovine calves, 6 to 12 months of age (*n* = 27)	1,0 mg/kg AFB1	600 mg/kg Syl	Improves ADFI, ADWG, hematological and serum biochemical parameters.	[153]
Bovine	Organic dairy herd	diet naturally contaminated with AFB1	10 g/d Syl, 30 g/d Syl phytosome	Lower emission of AFM1 in milk.	[154]
Rabbit	Female Oryctolagus cuniculi, 1 year and 2 months old. (*n* = 1)	300 mg/kg of feed AFB1	50 mg/kg/orally Syl	Continued use of silymarin effective in the treatment of toxic liver disease by AFB1.	[155]
**Grape pomace extracts**					
Broiler chickens	Ross-308, 1-day-old (*n* = 240)	400 mg/kg FB1	250 & 500 mg/kg GSE	Improves growth performance, serum immunoglobulin contents, serum biochemical contents, and enzyme activities in the liver, malondialdehyde content and total antioxidant capacity and glutathione peroxidase concentration within the liver and serum.	[176]
Broiler chickens	Cobb broilers, 1-day-old (*n* = 300)	1 mg/kg AFB1	250 & 500 mg/kg GSE	Reduces AFB1 residues in the liver. Increases serum immunoglobulins. Improves ADFI and ADG. Reduces ALT, AST, GGT, ALP. Increases total protein, albumin and globulin levels. Reduces serum and hepatic MDA and increases SOD, GSH-Px, GSH, GST and CAT. Reduces liver relative weight.	[177]
Broiler chickens	Ross-308, 1-day-old (*n* = 125)	76 ppb AFs	200mg/kg GSE	Increases antioxidant rates (GPx) and in the levels of antibodies (ND & IBD). Reduces AST and ALT, and MDA.	[178]

Japanese quails	Coturnix japónica, 14-day-old (*n* = 60)	1mg/kg AFB1	500 mg/kg GSPE	Alleviates hepatotoxicity as reflected in diminishing alanine transaminase, aspartate aminotransferase, alkaline phosphatase, lipid peroxidation, and raising TNF-*α* and IL-6 as pro-inflammatory cytokines. Increases glutathione peroxidase, catalase, and superoxide dismutase.	[180]
Swine	cross-bred TOPIGS-40 hybrid weaned piglets (*n* = 40)	62 ppb AFB1 + 479ppb OTA	5% of the mixture (1:1) of grape seed and sea buckthorn meal	Decreases CYP P450 gene expression, suggesting the decrease of bioactivation of these mycotoxins. Improves animal performance.	[181]
Swine	Pietrain/Duroc /Large-white weaned piglets, 4-week-old (*n* = 28)	2,2 μg/g FB1, 0,8 μg/g DON, 0,08 μg/g ZEN, 0,02 μg/g AFB1, & 0,02 μg/g OTA in the daily diet	50% red or white grape pomace in bolus	Reduces urinary mycotoxin biomarker of AFB 1 (67%) and ZEN (69%), demonstrating reduction of gastrointestinal absorption of mycotoxins	[182]
Swine	cross-bred TOPIGS-40 weaned piglets (*n* = 24)	320 ppb AFB1	8% of grape seed meal	Reduces inflammation and oxidative stress by decreasing IL-6 cytokine and lipid peroxidation and increasing CAT, SOD activities.	[183]
Swine	Crossbred weaned piglets (TOPIGS-40), 4-week-old (*n* = 24)	320 μg/kg feed AFB1	8% of grape seed meal	Reduces oxidative stress by increasing the activity of GPx and SOD and by decreasing lipid peroxidation. Decreases inflammatory markers IL-1β, IL-6, IL-8. Decreases NF-KB and MAPK protein quantification.	[184]
Swine	cross-bred TOPIGS-40 hybrid weaned piglets, (*n* = 24)	320 ppb AFB1	8% of grape seed meal	Enhances the antioxidant enzymes activities, decreases the pro-inflammatory cytokines and TBARS level, and ameliorates the growth performance	[185]
Swine	cross-bred TOPIGS-40 hybrid weaned piglets, (*n* = 24)	320 μg/kg feed AFB1	8% of grape seed meal	Increases CAT and SOD levels. Lowers lipid peroxidation. Overall improves antioxidant status.	[186]
Swine	cross-bred TOPIGS-40 hybrid weaned piglets, (*n* = 24)	320 g/kg feed AFB1	8% of grape seed meal	Increases body weight an lowers frequency of diarrea. Increases the relative abundance of phylum *Bacteroidetes* and *Proteobacteria*, while decreasing the *Firmicutes* abundance in the large intestine	[187]
Chinese sea bass	Lateolabrax maculatus (*n* = 480)	1 mg/kg AFB1	1 g/kg of tannins	Increases villus length and intestinal tight junction protein gene expression (ZO-1, Claudin-3 and Occludin). Ameliorates growth performance, serum parameters and intestinal morphology. Lowers abundance of *Pseudomonas.*	[188]

**Olive pomace extracts**					
Nile Tilapia	Oreochromis niloticus (*n* = 250)	4,8 mg/kg T-2 toxin	0.2% quercetin	Reduces the damage to fish liver and muscle by enhancing liver GST activity. Maintaines a stable WG similar to the control group.	[189]
**Orange peel extracts**					
Broiler chickens	Male, Arbor Acres, 1-day-old (*n* = 144)	100 µg/kg AFB1	200 mg/kg LYC	Ameliorates performance and intestinal integrity. Reduces AFB1-related oxidative and inflammatory status by increasing CAT, SOD, Nrf2 and GSH-Px expressions. Improves antioxidant activity in the intestine (GSH and GSH activities).	[242]
Broiler chickens	Arbor Acres, (*n* = 240)	100 µg/kg AFB1	400 mg/kg LYC	Lowers the heaptic activities of CYP1A1 and CYP2A6, and the hepatic concentrations of AFBO-DNA, ROS, MDA, 4-HNE, PC, and 8-OHdG. Increased hepatic GSH, GPx, CAT and GST activities.	[243]
Broiler chickens	Arbor Acres, 1-day-old (*n* = 192)	100 µg/kg AFB1	200 mg/kg LYC	Decreases the feed conversion ratio, yellowness and shear force, and the content of PC and hydrogen peroxide. Increases the ADG, breast muscle redness, and breast muscle's ability to clear $ABTS^{\cdot+}$. Alleviates the negative impacts of AFB1 on the growth performance, meat quality, and antioxidant capacity of breast muscle in broilers.	[244]
Swine	Porcine IPEC-J2 cells	1 µ g/mL DON	10 µM LYC	Lyc alleviates cell damage and decreases cell apoptotic rate. Lyc down-regulates the content of ROS and restores antioxidant enzyme activity. Lyc may activate the oxidative phosphorylation (OXPHOS) to improve mitochondrial function.	[245]
Swine	piglet sertoli cells	ZEN (5, 10, 15, 20, 25, 30 µM)	LYC (10, 20, 30, 40, 50, 60 µM)	Improves the cell survival rate, promotes the expression of Nrf2 in the nucleus, upregulates the relative mRNA expression of HO-1 and GPX1, increases the activity of antioxidant enzymes, and reduces the levels of MDA and ROS. Decreases apoptosis rate and autophagy.	[246]
